# PANEV: an R package for a pathway-based network visualization

**DOI:** 10.1186/s12859-020-3371-7

**Published:** 2020-02-06

**Authors:** Valentino Palombo, Marco Milanesi, Gabriella Sferra, Stefano Capomaccio, Sandy Sgorlon, Mariasilvia D’Andrea

**Affiliations:** 1 Dipartimento Agricoltura, Ambiente e Alimenti, Università degli Studi del Molise, 86100 Campobasso, Italy; 2 Department of Support, Production and Animal Health, School of Veterinary Medicine, São Paulo State University, Araçatuba, São Paulo 16050-680 Brazil; 3 Istituto di Zootecnica, Università Cattolica del Sacro Cuore, 29122 Piacenza, Italy; 4 Dipartimento di Bioscienze e Territorio, Università degli Studi del Molise, 86090 Pesche, IS Italy; 5 Dipartimento di Medicina Veterinaria, Università di Perugia, 06126 Perugia, Italy; 6 Dipartimento di Scienze Agrarie ed Ambientali, Università degli Studi di Udine, 33100 Udine, Italy

**Keywords:** Molecular pathways, Pathway visualization, Genomic and transcriptomic analysis, Data mining, KEGG

## Abstract

**Background:**

During the last decade, with the aim to solve the challenge of post-genomic and transcriptomic data mining, a plethora of tools have been developed to create, edit and analyze metabolic pathways. In particular, when a complex phenomenon is considered, the creation of a network of multiple interconnected pathways of interest could be useful to investigate the underlying biology and ultimately identify functional candidate genes affecting the trait under investigation.

**Results:**

PANEV (PAthway NEtwork Visualizer) is an R package set for gene/pathway-based network visualization. Based on information available on KEGG, it visualizes genes within a network of multiple levels (from 1 to *n*) of interconnected upstream and downstream pathways. The network graph visualization helps to interpret functional profiles of a cluster of genes.

**Conclusions:**

The suite has no species constraints and it is ready to analyze genomic or transcriptomic outcomes. Users need to supply the list of candidate genes, specify the target pathway(s) and the number of interconnected downstream and upstream pathways (levels) required for the investigation. The package is available at https://github.com/vpalombo/PANEV.

## Background

Thanks to advancements in high-throughput techniques and simultaneous reduction in the associated costs, large scale ‘omics’ studies are now common. These studies enable the generation of a huge amount of biological data [1] and pose to the researchers the challenge of data mining, rather than data production. The key result of genomic (e.g. genome-wide association study) or transcriptomic analysis (e.g. gene expression profiling) is a long list of statistically significant genes that, supposedly, contribute to the studied phenomenon. The subsequent step, after the exclusion of false positive signals, is to extract meaning from them, in order to provide insights into the underlying complex biology of the phenotype under study [2]. One common strategy to reduce the complexity of this challenge is grouping the genes into smaller sets of related ones, for example, sharing the same biological processes (i.e. pathway). This pathway-based approach [3] has become popular during the last years [4] and is, de facto*,* the standard for the post-omics analysis of high-throughput experiments [5].

Pathway analysis and visualization tools are now successfully and routinely applied to gene expression and genetic data analyses and they represent a support key to understand biological systems [6–11]. In this regard, pathway-based approaches are particularly useful when complex phenomena, with a quantitative inheritance, are under study [12]. Compared with an individual gene-based approach, the strategy to create a network of multiple related pathways and genes of interest is more suitable to explore the biology of complex traits and identify functional candidate genes [13, 14]. The increase in the availability of repositories based on hierarchical and/or functional classification of terms helped in this exploration [15]. Many web resources are now available, providing access to many thousands of pathways (see http://pathguide.org/). Among the others, a prominent reference repository, constantly updated, is the Kyoto Encyclopedia of Genes and Genomes (KEGG) [16]. KEGG is a bioinformatics resource that maps genes to specific pathways and summarizes them into one connected and manually curated metabolic network.

Here, we introduce the PANEV (PAthway NEtwork Visualizer) R package that represents an easy way to visualize genes into a network of pathways of interest. The novelty of PANEV visualization relies on the creation of a customized network of multiple interconnected pathways, considering *n* levels (as required by the user) of upstream and downstream ones. The network is created using KEGG information [16]. As far as we know, no other KEGG visualization tool [6–8] provides such a feature that may help to identify functional candidate genes among the list of provided ones. PANEV has also features that are rarely simultaneously available in other pathway visualization tools [7, 17, 18]. In particular, (i) it handles data from all the species included in KEGG databases, (ii) it provides fully accessible graphics through an interactive visualization module that allows the user to easily navigate the generated network, (iii) it is easy to be integrated with other pathway analysis or gene set enrichment analysis tools.

## Implementation

The package is specifically designed for post-genomic and post-transcriptomic data visualization. The rationale of graphical visualization performed by PANEV is to identify candidate genes taking into account a network of ‘functionally’ related pathways. The ‘functional’ network is created considering a set of main pathways of interest (first level pathways - 1 L), chosen by the user since known to be involved in the phenomenon under study, and multiple levels of interconnected pathways, added by PANEV on the basis of information retrieved on KEGG database [16, 19]. Each level considers the pathways connected with the previous ones. These pathways represent de facto the upstream and downstream pathways without reconstructing the direction of relations in the PANEV graphical output. Once the ‘functional’ network is created, PANEV visualizes the genes among the list of those provided by the user. The network visualization is generated in html output format using the visNetwork R package (https://cran.r-project.org/web/packages/visNetwork), which guarantees fully interactive graphs.

### Package installation and functionality

The package PANEV v.1.0 is available at https://github.com/vpalombo/PANEV. It can be easily downloaded and installed in any R session (R ≥ 3.5.0) using the install_github(“vpalombo/PANEV”) function, from the devtools package (https://cran.r-project.org/package=devtools). The tool requires other libraries automatically uploaded along with the package. Once installed, PANEV can be loaded in the R environment with the library(‘PANEV’) command.

**Fig. 1:**
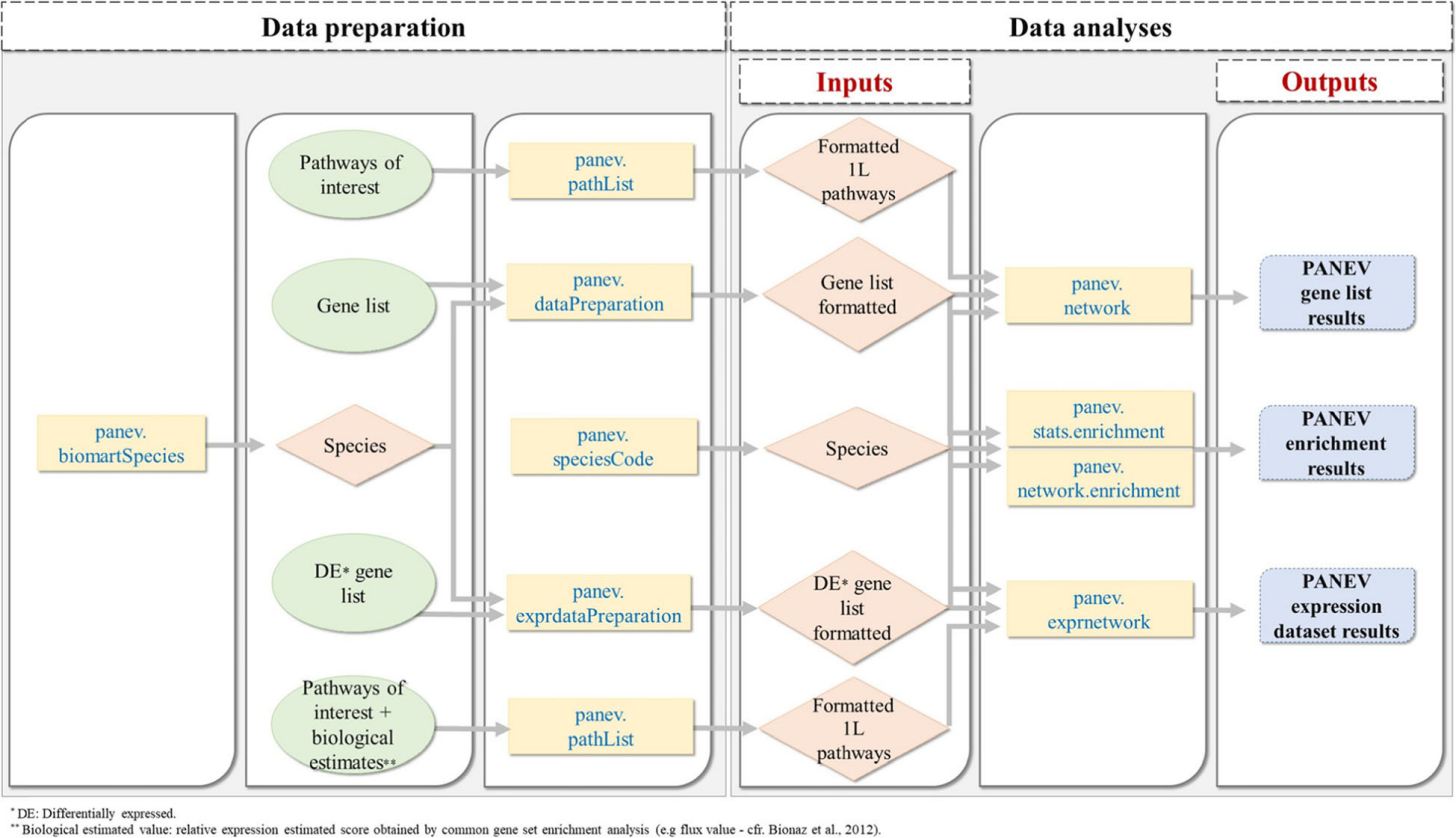
The general architecture of the workflow of the PANEV package and schematic illustration of the main functions. The yellow rectangles represent the PANEV functions. The green circles represent the input data lists, in particular gene or pathway lists. The red diamonds represent the output from the PANEV ‘data preparation’ functions. The blue rectangles represent the final PANEV outcomes

PANEV package functions could be divided in two steps: data preparation and data analyses (Fig. 1). The first step helps to prepare a properly formatted list of genes and 1 L pathways, as well as to obtain all mandatory information required to run PANEV analyses. The second step performs data analysis and visualization.

Since PANEV interrogates KEGG databases [16], an internet connection is required. Access to KEGG repositories has specific copyright conditions (https://www.kegg.jp/kegg/legal.html). PANEV uses the KEGGREST package (https://bioconductor.org/packages/release/bioc/html/KEGGREST.html) functions to download individual pathway graphs and data files through API or HTTP access, which is freely available for academic and non-commercial uses.

Trial datasets are available in the package and can be stored in the working directory using the panev.example() command.

### Data preparation

To enhance user experience, data preparation functions are available. In particular, PANEV provides two specific functions, panev.dataPreparation() and panev.exprdataPreparation(), to obtain a proper input data format from a simple gene list or an expression gene list, respectively. Their correct performance depends on the availability of biomaRt [20] data access for a specific species of interest. The list of all the available species for biomaRt annotation can be retrieved by the panev.biomartSpecies() command.

Along with the correct KEGG organism code, obtainable with the panev.speciesCode() function, a list of main pathways of interest (1 L) is mandatory to properly run PANEV. The list of all KEGG investigable pathways can be retrieved by the panev.pathList() function. In the case of analysis on an expression gene list, the 1 L pathway(s) must be provided with a pathway expression estimated score(s). The pathway estimated score can be obtained by using common gene set enrichment analysis or over-represented approach analysis [21] (e.g. flux value [22], as in the trial data).

### Data analyses and visualization

**Fig. 2:**
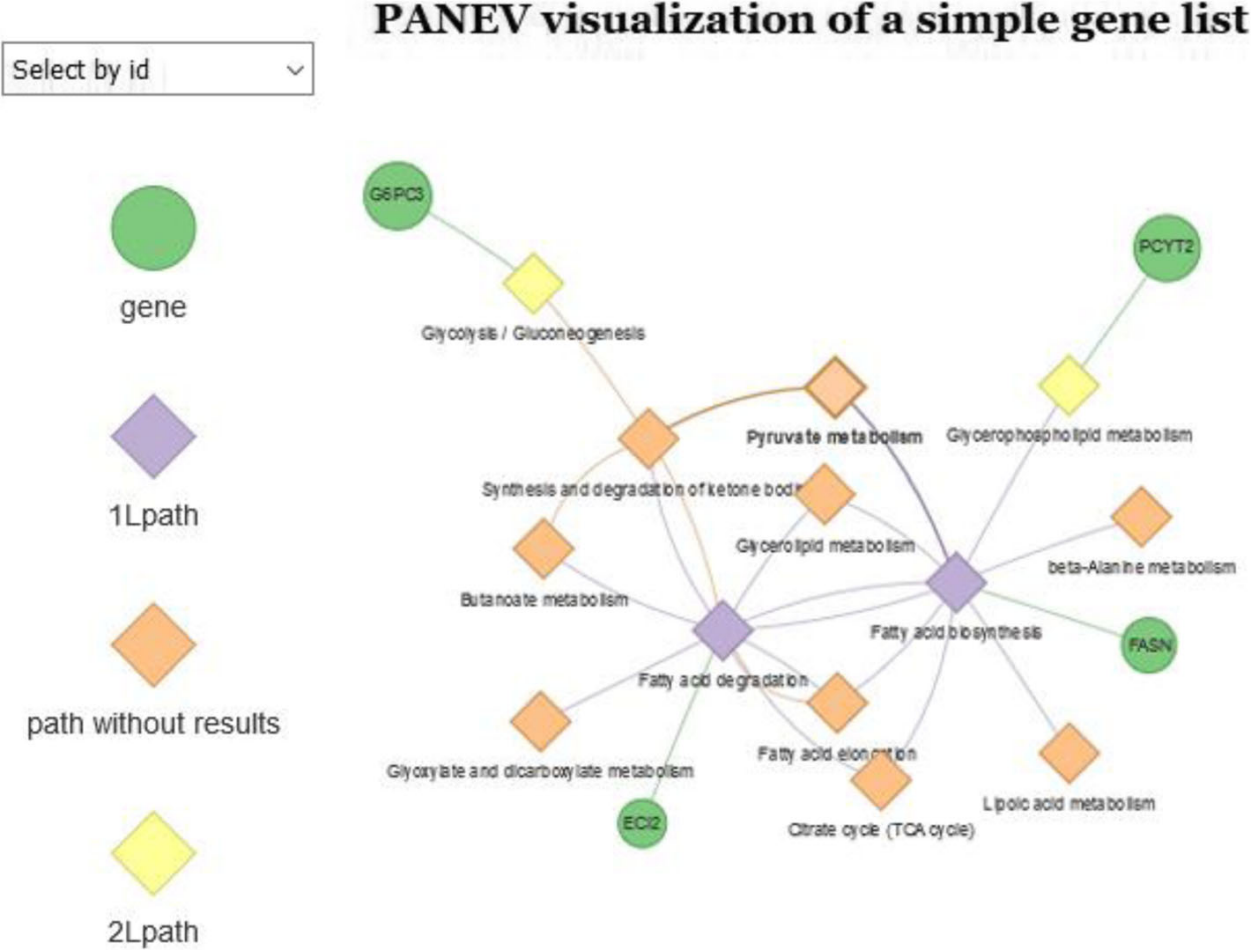
An example of the gene/pathway network visualization of PANEV results. The green circles represent the candidate genes connected with the pathways in the network. The violet diamonds represent the first-level (1 L) pathways. The yellow diamonds represent the second-level (2 L) pathways. The orange diamonds represent the pathways belonging to the network but without connection with any candidate gene. The diagram is saved in ‘.html’ format

The panev.network() function allows performing PANEV visualization on a simple gene list (e.g. genomic analysis). The function requires (i) a properly formatted gene list, (ii) a vector of 1 L pathways, (iii) the KEGG organism code and (iv) the number of levels to investigate (from 1 to *n*), which represents how many levels of interconnected (upstream/downstream) pathways will be explored. If the argument is set as 1, only 1 L pathway(s) will be used to create the network. The panev.network() function firstly creates a framework of interconnected pathways, starting from 1 L pathways, and it subsequently highlights the genes from the input gene list inside the generated ‘functional’ network. The function creates an interactive graph, summarizing the genes/pathways network results and enabling the selection and magnification of a specific node (Fig. 2). Moreover, it generates one text file containing the tabular results of the highlighted genes for each level analyzed.

**Fig. 3:**
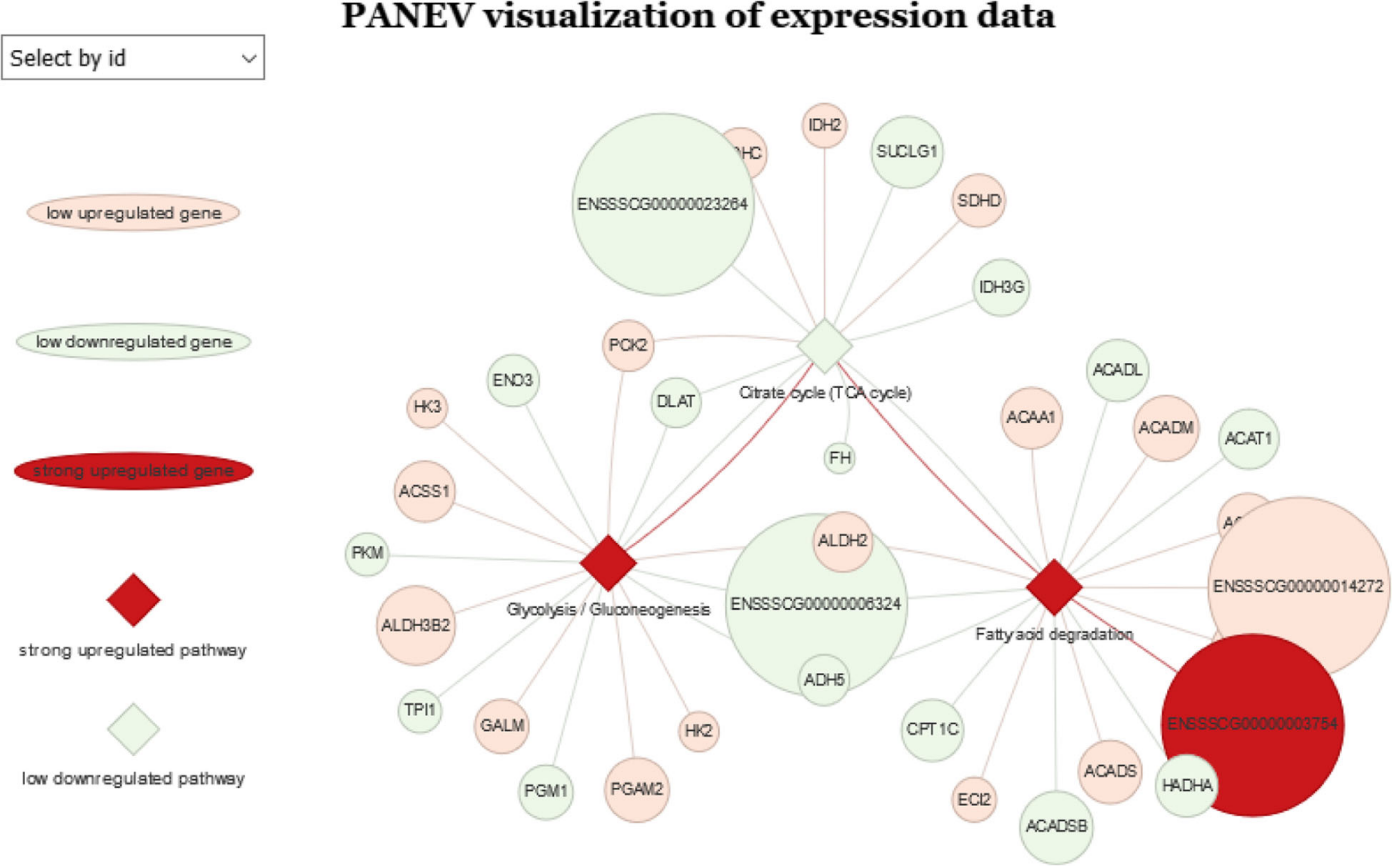
An example of the ‘.html’ file with the network-based visualization of PANEV results considering an expression dataset. The circles represent the genes colored based on their fold change (FC) values. The diamonds represent the pathways of interest colored based on their expression estimated scores

For gene expression datasets, PANEV takes into account any possible connection among a custom list of pathways of interest and a list of differentially expressed genes (DEGs). The dedicated function is panev.exprnetwork() that requires (i) a properly formatted DEG list with fold change (FC) values and *p-values*, (ii) a properly formatted list of pathways with expression estimated scores, (iii) the KEGG organism code and (iv) a *p-value* cut-off for filtering subsets of genes in the DEG list. The function generates the interactive diagram visualization of the gene/pathway network (Fig. 3). Gene/pathway nodes are colored according to their gene FC and pathway expression estimated scores, following the classification reported in Table 1.

**Table 1:** Summary of node (genes and pathways) color classification in the network graph visualization obtained with panev.exprnetwork() function. The upregulated genes/pathways are reported using a red scale, from light red (low) to dark red (strong). The downregulated genes/pathways are reported using a green scale, from light green (low) to dark green (strong)

Gene / Pathway classification	Gene fold change (FC) / Pathway expression score value
low upregulated/downregulated	< 25% of top up/downregulated gene/pathway value
moderate upregulated/downregulated	≥25% and < 50% of top up/downregulated gene/pathway value
high upregulated/downregulated	≥ 50% and < 75% of top up/downregulated gene/pathway value
strong upregulated/downregulated	≥ 75% of top up/downregulated gene/pathway value

PANEV also provides the ancillary functions panev.stats.enrichment() and panev.network.enrichment() to perform a gene enrichment analysis based on a hypergeometric test (one-sided Fisher exact test), as described by Simoes and Emmert-Streib [23]. In particular, while the former function allows the user to search against the default KEGG database, the latter computes the pathway enrichment of the genes highlighted by PANEV using the pathways generated in the network as a background. The results are text files containing enrichment analysis outcomes and tables with gene/pathway occurrences. For each pathway, a *p-value* is calculated to estimate its probability of over-representation [23].

## Results and discussion

To evaluate and validate the usefulness of PANEV, we used a publicly available dataset on human type 1 diabetes mellitus (T1DM) [24]. In the reference study, the authors carried out a gene-based genome-wide association study (GWAS) and identified 452 significant genes. Among these, 171 genes were newly associated with T1DM and 53 out of 171 were supported by replication or differential expression studies. In particular, four non-HLA (human leukocyte antigen) genes (*RASIP1*, *STRN4*, *BCAR1* and, *MYL2*) and three HLA genes (*FYN*, *HLA-J* and *PPP1R11*) represent the main result discussed by the authors, since validated by both the replication and the differential expression studies.

To verify the possible contribution of the PANEV tool to the identification of functional candidate genes, we performed PANEV analysis considering the list of 171 newly identified genes. The validation datasets are available in the package and can be stored in working directory using the panev.example(type = “validation”) command.

After data preparation, 5 out of 171 genes having no corresponding entrez ID were excluded from the further analyses. Considering the complexity of the investigated trait, PANEV was performed up to the third level of interaction [25]. The ‘*Type I diabetes mellitus*’ (map04940), ‘*Insulin resistance*’ (map04931) and ‘*AGE-RAGE signaling pathway in diabetic complications*’ (map04933) pathways were chosen as 1 L pathways, since clearly associated in the literature with T1DM [26, 27]. A summary of PANEV results is reported in Additional files 1 and 2.

Fifteen out of 166 genes were highlighted at different levels as functional candidates by PANEV (Additional file 1). In particular, PANEV identified 4 out of 7 genes mainly discussed in reference study: *PTPN11* at 1 L, *FYN* at 2 L, *BCAR1* and *MYL2* at 3 L. The three genes (*RASIP1*, *STRN4* and, *HLA-J*) not detected by PANEV are in KEGG databases but not assigned yet to any pathway. It is interesting to note that PANEV identified also other well-known genes (*ITPR3*, *BAK1* and *IL10* at 2 L; *HMGB1* and *MICA* at 3 L), already associated with T1DM [28–31] but not discussed by Qui and colleagues [24], since they were confirmed only by the differential expression or replication studies. Furthermore, PANEV highlighted other genes reported in the literature as being associated with the susceptibility to T1DM disease but not discussed in the reference study [24], since not confirmed neither by the differential expression nor by replication studies. In particular, *CDK2* [32], *SMAD7* [33], *STAT4* [34], *BCL2A1* [35] and *RXRB* [36] were shown at 2 L, whereas *MADCAM1* [37] at 3 L. It is worth to note that, except for *CDK2,* all genes mentioned above refer to researches conducted before the reference study [24]. Simultaneously, it must be observed that 138 genes were excluded by PANEV during the analysis, because (i) assigned to pathways not included in the three investigated levels (~ 8%), (ii) not present in KEGG databases (~ 39%), or (iii) not assigned yet to any pathway (~ 48%). The first point is suggestive of PANEV capability to discriminate false positive among the list of provided genes. The last two points clearly represent the main limitations of PANEV due to KEGG’s incomplete information. A comparison among PANEV results and reference study [24] is reported in Additional file 3.

Accordingly to the reference study [24], we also performed the enrichment analysis of KEGG pathways considering the 452 genes identified by the authors. The results obtained by PANEV enrichment function showed an over-representation of immune diseases and immune system pathways (Additional file 4), in line with Qiu et al. [24] outcomes.

PANEV was already applied by Palombo and colleagues on genes significantly associated with milk fatty acid profiles in Italian Simmental and Holstein breeds [38]. A total of 47 and 165 significant positional candidate genes were detected in Italian Simmental and Holstein breeds, respectively. Among these genes, PANEV highlighted three lipogenic genes well described in the literature: *SCD*, *DGAT* and *FASN*. Furthermore, fifteen new functional candidate genes directly or indirectly involved in ‘Lipid metabolism’ pathways were identified.

In summary, PANEV offers advantages in terms of timesaving and speeding up data mining. In particular, candidate genes with strong literature support could be rapidly identified without any validation study. These candidate genes could be quickly subjected to the further study phases (such as in vivo validation). Moreover, gene and pathway connections could be easily identified using the diagram visualization and this information might be interesting to discuss in manuscript drafting. About the putative candidate genes not highlighted by PANEV, these could be retrieved using conventional methods, such as deeper literature research or in silico validation, which remain more time consuming and costly.

## Conclusion

PANEV is a package entirely built in R and represents a novel and useful visualization tool to reduce the complexity of the high-throughput data mining challenge and identify candidate genes. PANEV creates customized gene/pathway network graphs considering a list of candidate genes and multiple levels of interconnected (upstream and downstream) pathways of interest. This helps the interpretation of genomic and transcriptomic analysis outcomes, in particular when complex biological phenomena are investigated.

The contribution of the PANEV tool could be significant not only for well-annotated species (i.e. *Homo sapiens*, *Mus musculus*) but also for all the organisms available in KEGG databases. Although KEGG is a popular and constantly updated database, the lack or incomplete information could represent the main PANEV disadvantage, as for other KEGG-based tools. The effectiveness of PANEV analysis in terms of result coherency was confirmed by the validation study. In particular, PANEV produces timesaving advantages, pointing the user to genes that are biologically involved with the investigated trait.

## Availability and requirements

Project name: PANEV.

Project home page: https://github.com/vpalombo/PANEV

Operation systems: Platform independent.

Programming language: R (> = 3.5.0).

License: Artistic-2.0.

Restrictions to use by non-academics: Yes (i.e. KEGG subscription).

## Supplementary information

**Additional file 1.** Summary of the tabular result obtained by PANEV using the data from Qui et al. (2014) study and considering three levels of interactions ‘Type I diabetes mellitus’, ‘Insulin resistance’, and ‘AGE-RAGE signaling pathway in diabetic complications’ as 1 L pathways

**Additional file 2.** Screenshot of network-based visualization result obtained by PANEV using the data from Qui et al. (2014) study and considering three levels for the investigation. The violet diamonds represent the first-level (1 L) pathways (in this case: ‘Type I diabetes mellitus’, ‘Insulin resistance’, and ‘AGE-RAGE signaling pathway in diabetic complications’) connected with candidate genes. The yellow and the blue diamonds represent the second (2 L) and third-levels (3 L) pathways connected with candidate genes, respectively. The orange diamonds represent the pathways belonging to the network without connection with any candidate gene

**Additional file 3.** Comparison between PANEV and reference study results (Qiu et al., 2014)

**Additional file 4.** PANEV enrichment result of KEGG pathways considering the 452 genes identified by the Qiu et al. (2014)

## Data Availability

The data that support the findings of this study, as well as reproducible examples, are available at https://github.com/vpalombo/PANEV/tree/master/vignettes and were generated from the following study: Qiu Y-H, Deng F-Y, Li M-J, Lei S-F. Identification of novel risk genes associated with type 1 diabetes mellitus using a genome-wide gene-based association analysis. J Diabetes Investig. 2014. doi:10.1111/jdi.12228

## Abbreviations

1 L: First level pathway
2 L: Second level pathway
3 L: Third level pathway
AGE: Advanced glycation end products
BAK1: BCL2 Antagonist/Killer 1
BCAR1: Breast Cancer Anti-Estrogen Resistance 1
BCL2A1: BCL2 Related Protein A1
CDK2: Cyclin Dependent Kinase 2
DEG: Differentially expressed gene
DGAT: Diacylglycerol O-Acyltransferase
FASN: Fatty Acid Synthase
FC: Fold change
FYN: FYN Proto-Oncogene, Src Family Tyrosine Kinase
GWAS: Genome-wide association study
HLA: Human leukocyte antigen
HMGB1: High Mobility Group Box 1
IL10: Interleukin 10
ITPR3: Inositol 1,4,5-Trisphosphate Receptor Type 3
KEGG: Kyoto Encyclopedia of Genes and Genomes
MADCAM1: Mucosal Vascular Addressin Cell Adhesion Molecule 1
MICA: MHC Class I Polypeptide-Related Sequence A
MYL2: Myosin Light Chain 2
PANEV: Pathway Network Visualizer
PPP1R11: Protein Phosphatase 1 Regulatory Inhibitor Subunit 11
RAGE: Receptor for advanced glycation end products
RASIP1: Ras Interacting Protein 1
RXRB: Retinoid X Receptor Beta
SCD: Stearoyl-CoA Desaturase
SMAD7: Mothers Against Decapentaplegic Homolog 7
STAT4: Signal Transducer And Activator Of Transcription 4
STRN4: Striatin 4
T1DM: type 1 diabetes mellitus
