# Supplementary material for: PANEV: an R package for a pathway-based network visualization
Source: BMC Bioinformatics. 2020 Feb 6;21:46. doi: 10.1186/s12859-020-3371-7 (PMC7006390; doi:10.1186/s12859-020-3371-7)
Supplement: Supplementary file 1 — Additional file 1. Summary of the tabular result obtained by PANEV using the data from Qui et al. (2014) study and considering three levels of interactions ‘Type I diabetes mellitus’, ‘Insulin resistance’, and ‘AGE-RAGE signaling pathway in diabetic complications’ as 1 L pathways [file 12859_2020_3371_MOESM1_ESM.docx]

| ***Level investigated*** | ***KEGG pathway*** | ***Gene*** |
| --- | --- | --- |
| 1L | Insulin resistance | *PTPN11* |
| 2L | PI3K-Akt signaling pathway | *CDK2* |
|  | Apoptosis | *ITPR3, BAK1, BCL2A1* |
|  | T cell receptor signaling pathway | *FYN, IL10* |
|  | Calcium signaling pathway | *ITPR3* |
|  | Jak-STAT signaling pathway | *STAT4, IL10, PTPN11* |
|  | Cell cycle | *CDK2* |
|  | TGF-beta signaling pathway | *SMAD7* |
|  | Adipocytokine signaling pathway | *RXRB, PTPN11* |
| 3L | Chemokine signaling pathway | *BCAR1* |
|  | NF-kappa B signaling pathway | *BCL2A1* |
|  | FoxO signaling pathway | *CDK2, IL10* |
|  | Phosphatidylinositol signaling system | *ITPR3* |
|  | Cytokine-cytokine receptor interaction | *IL10* |
|  | p53 signaling pathway | *CDK2* |
|  | Autophagy - animal | *HMGB1* |
|  | Protein processing in endoplasmic reticulum | *BAK1* |
|  | Focal adhesion | *BCAR1, FYN, MYL2* |
|  | Cell adhesion molecules (CAMs) | *MADCAM1* |
|  | Vascular smooth muscle contraction | *ITPR3* |
|  | Natural killer cell mediated cytotoxicity | *MICA, FYN, PTPN11* |
|  | Long-term potentiation | *ITPR3* |
|  | Long-term depression | *ITPR3* |
|  | Renin secretion | *ITPR3* |
|  | Aldosterone synthesis and secretion | *ITPR3* |
|  | Regulation of actin cytoskeleton | *MYL2, BCAR1* |
