# Supplementary figures and images for: PANEV: an R package for a pathway-based network visualization
Source: BMC Bioinformatics. 2020 Feb 6;21:46. doi: 10.1186/s12859-020-3371-7 (PMC7006390; doi:10.1186/s12859-020-3371-7)

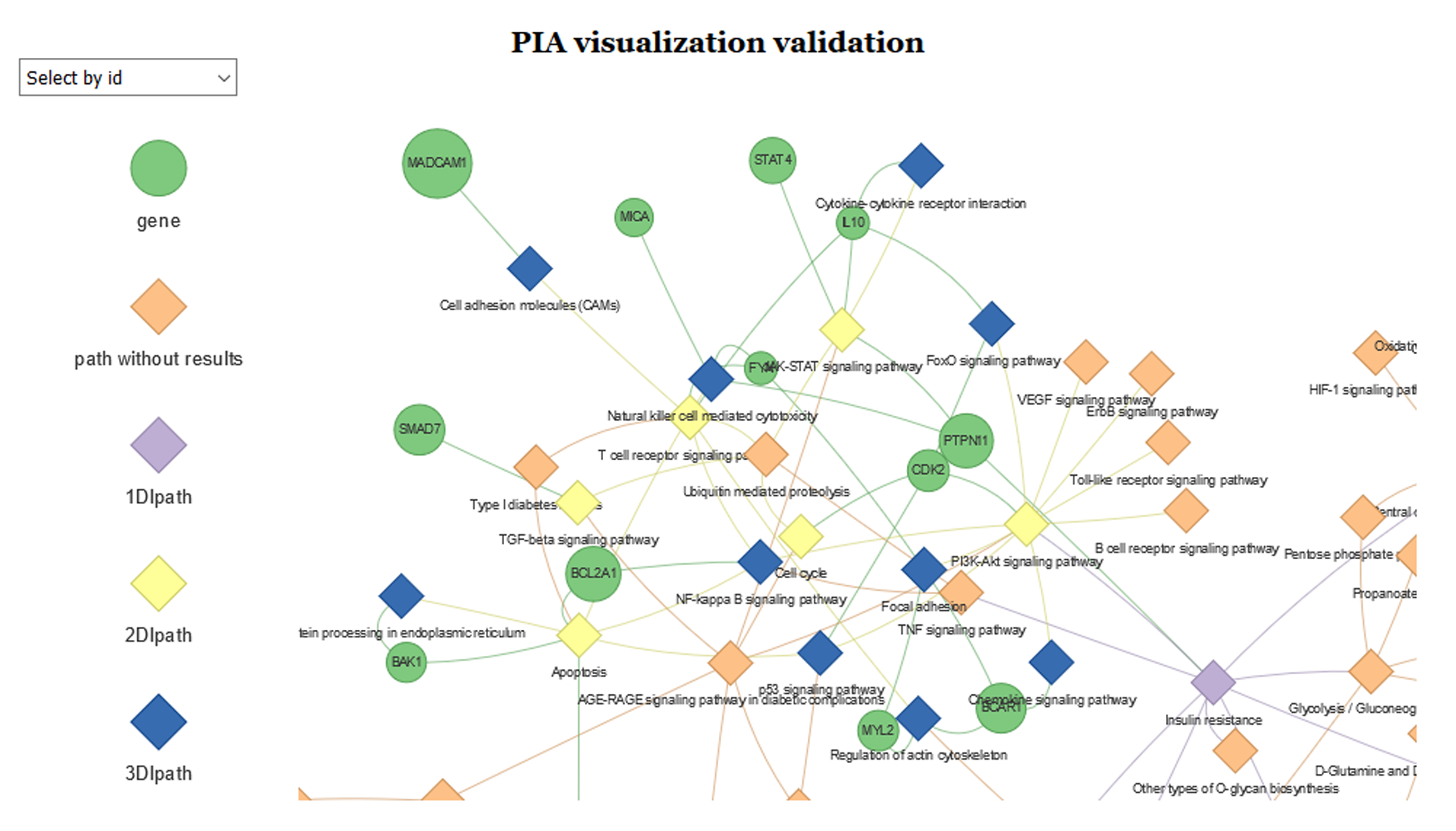

Supplement: Supplementary file 2 — Additional file 2. Screenshot of network-based visualization result obtained by PANEV using the data from Qui et al. (2014) study and considering three levels for the investigation. The violet diamonds represent the first-level (1 L) pathways (in this case: ‘Type I diabetes mellitus’, ‘Insulin resistance’, and ‘AGE-RAGE signaling pathway in diabetic complications’) connected with candidate genes. The yellow and the blue diamonds represent the second (2 L) and third-levels (3 L) pathways connected with candidate genes, respectively. The orange diamonds represent the pathways belonging to the network without connection with any candidate gene [file 12859_2020_3371_MOESM2_ESM.docx]
