# Supplementary material for: PANEV: an R package for a pathway-based network visualization
Source: BMC Bioinformatics. 2020 Feb 6;21:46. doi: 10.1186/s12859-020-3371-7 (PMC7006390; doi:10.1186/s12859-020-3371-7)
Supplement: Supplementary file 3 — Additional file 3. Comparison between PANEV and reference study results (Qiu et al., 2014) [file 12859_2020_3371_MOESM3_ESM.docx]

| ***Genes* highlighted by PANEV and consistent with main result in reference*** ***study*** |
| --- |
| *PTPN11, BCAR1, MYL2* and *FYN* |
| * among the 7 genes validated both in replication and differential expression studies (Qiu et al., 2014) |
| ***Genes* highlighted by PANEV, reported in literature as being associated to the susceptibility to T1DM disease, but not discussed in reference study*** |
| *ITPR3* (Qu et al., 2008), *BAK1* (Qiu et al., 2014), *IL10* (Hong et al., 2009), *HMGB1* (Zhang et al., 2010), *MICA* (Park et al., 2001), *CDK2* (Kim et al., 2017), *MADCAM1* (Phillips et al., 2005), *STAT4* (Bi et al., 2013), *BCL2A1* (Beyan et al., 2010), *SMAD7* (Chen et al., 2011), *RXRB* (Rajsbaum et al., 2002) |
| * among the 53 genes validated in replication studies or in differential expression studies (Qiu et al., 2014) |
| ***Genes* not highlighted by PANEV, since falling inside no-investigated pathways*** |
| *BRAP*, *FUT2, GNS, HIPK1, NUPR1, OR2B3, HIST1H4E, HIST1H2BF, OR2B3, OR2B6, OR2J2, OR5V1, SULT1A1* |
| * among 166 out of 171 newly genes in reference study (Qiu et al., 2014) |
| ***Genes* not highlighted by PANEV since not yet assigned to any KEGG pathways*** |
| *ADAD1*, *ASCL2*, *ATF7IP*, BTN3A3, C6orf227, *CABP1, CCDC101, CEACAM7, CRYZL1, DEXI, ETF1P1, FAM46B, FAP, GCA, GGNBP1, GNL1, GP2, GUSBL1, HIST1H1A, HIST1H1T, HIST1H2BD, HIST1H3H, HIST1H4F, HIST1H4G, HIST1H4PS1, HLA-J, HORMAD2, IKZF1, IKZF3, KIAA0528, KIFC1, KRT222, LHX9, LOC144481, LOC284749, MAMSTR, MICG, MIR548H3, MIR600, MPZL3, NCAPD2, NSL1, OLFML3, OR12D1P, ORMDL3, PHF1, PLBD1, PLEKHA1, PPP1R10, PPP1R11, PRR3, PRSS16, RASIP1, RING1, SBK1, SCGN, SLC17A1, SLC17A2, SLC17A3, SLC17A4, SPRR2E, STRN4, TAPBPL, TMEM129, TMEM170A, VPS52, ZBTB9, ZNF192, ZNF274, ZNF322A, ZNF323, ZPBP2, ZZEF1* |
| * among 166 out of 171 newly genes in reference study (Qiu et al., 2014) |
| ***Genes* not highlighted by PANEV since had no corresponding gene in KEGG databases*** |
| *GPR89P, HCG2P8, HCG4P3, HCG4P4, HCG4P9, HCGVIII-2, HCP5P2, LOC100127934, LOC100128077, LOC100128588, LOC100129387, LOC100130535, LOC100133214, LOC100270746, LOC100288130, LOC100506705, LOC100506979, LOC100507085, LOC340192, LOC402641, LYPLA2P1, OR2E1P, OR2U1P, OR2W6P, RPLP2P1, RPS10P1, RSPH1, SUMO2P, TRAJ57, TRAJ58, TRDD1, TRDD2, TRIM26, TRIM27, TRMEP1, TRNAA12, TRNAA19, TRNAA38, TRNAA40, TRNAA41, TRNAA5, TRNAF3, TRNAI1, TRNAI2, TRNAK43P, TRNAK8, TRNAL12, TRNAL47P, TRNAM15, TRNAM16, TRNAM4, TRNAM8, TRNAR10, TRNAS7, TRNAT11, TRNAT16, TRNAT7, TRNAV15, TRNAV27, TRNAV7, TRNAW2, TRNAY7, TRNAY8, UBD, VN1R14P* |
| * among 166 out of 171 newly genes in reference study |

***References***

Beyan, H., R.C. Drexhage, L. van der Heul Nieuwenhuijsen, H. de Wit, R.C. Padmos, N.C. Schloot, H.A. Drexhage, and R.D. Leslie. 2010. Monocyte gene-expression profiles associated with childhood-onset type 1 diabetes and disease risk: a study of identical twins. Diabetes 59:1751–1755. doi:10.2337/db09-1433.

Bi, C., B. Li, Z. Cheng, Y. Hu, Z. Fang, and A. Zhai. 2013. Association study of STAT4 polymorphisms and type 1 diabetes in Northeastern Chinese Han population. Tissue Antigens 81:137–140. doi:10.1111/tan.12057.

Chen, H.Y., X.R. Huang, W. Wang, J.H. Li, R.L. Heuchel, A.C.K. Chung, and H.Y. Lan. 2011. The protective role of Smad7 in diabetic kidney disease: mechanism and therapeutic potential. Diabetes 60:590–601. doi:10.2337/db10-0403.

Hong, E.-G., H.J. Ko, Y.-R. Cho, H.-J. Kim, Z. Ma, T.Y. Yu, R.H. Friedline, E. Kurt-Jones, R. Finberg, M.A. Fischer, E.L. Granger, C.C. Norbury, S.D. Hauschka, W.M. Philbrick, C.-G. Lee, J.A. Elias, and J.K. Kim. 2009. Interleukin-10 prevents diet-induced insulin resistance by attenuating macrophage and cytokine response in skeletal muscle. Diabetes 58:2525–2535. doi:10.2337/db08-1261.

Kim, S.Y., J.-H. Lee, M.J. Merrins, O. Gavrilova, X. Bisteau, P. Kaldis, L.S. Satin, and S.G. Rane. 2017. Loss of cyclin dependent kinase 2 in the pancreas links primary β-cell dysfunction to progressive depletion of β-cell mass and diabetes. J. Biol. Chem. jbc.M116.754077. doi:10.1074/jbc.M116.754077.

Park, Y., H. Lee, C.B. Sanjeevi, and G.S. Eisenbarth. 2001. MICA polymorphism is associated with type 1 diabetes in the Korean population. Diabetes Care 24:33–38.

Phillips, J.M., K. Haskins, and A. Cooke. 2005. MAdCAM-1 is needed for diabetes development mediated by the T cell clone, BDC-2·5. Immunology 116:525–531. doi:10.1111/j.1365-2567.2005.02254.x.

Qiu, Y.-H., F.-Y. Deng, M.-J. Li, and S.-F. Lei. 2014. Identification of novel risk genes associated with type 1 diabetes mellitus using a genome-wide gene-based association analysis. J Diabetes Investig 5:649–656. doi:10.1111/jdi.12228.

Qu, H.-Q., L. Marchand, A. Szymborski, R. Grabs, and C. Polychronakos. 2008. The association between type 1 diabetes and the ITPR3 gene polymorphism due to linkage disequilibrium with HLA class II. Genes Immun. 9:264–266. doi:10.1038/gene.2008.12.

Rajsbaum, R., D. Fici, D.A. Boggs, P.A. Fraser, P.O. Flores-Villanueva, and Z.L. Awdeh. 2002. Linkage disequilibrium between HLA-DPB1 alleles and retinoid X receptor β haplotypes. Human Immunology 63:771–778. doi:10.1016/S0198-8859(02)00427-5.

Zhang, S., J. Zhong, P. Yang, F. Gong, and C.-Y. Wang. 2010. HMGB1, an innate alarmin, in the pathogenesis of type 1 diabetes. Int J Clin Exp Pathol 3:24–38.
