# Supplementary material for: PANEV: an R package for a pathway-based network visualization
Source: BMC Bioinformatics. 2020 Feb 6;21:46. doi: 10.1186/s12859-020-3371-7 (PMC7006390; doi:10.1186/s12859-020-3371-7)
Supplement: Supplementary file 4 — Additional file 4. PANEV enrichment result of KEGG pathways considering the 452 genes identified by the Qiu et al. (2014) [file 12859_2020_3371_MOESM4_ESM.docx]

| ***Pathway name*** | ***Pathway ID*** | ***pvalue*** | ***adjusted p-value ***** |
| --- | --- | --- | --- |
| Systemic lupus erythematosus | path:hsa05322 | 1.94E-22 | 6.40E-20 |
| Allograft rejection | path:hsa05330 | 4.25E-15 | 7.01E-13 |
| Antigen processing and presentation | path:hsa04612 | 1.62E-14 | 1.75E-12 |
| Autoimmune thyroid disease | path:hsa05320 | 2.13E-14 | 1.75E-12 |
| Type I diabetes mellitus | path:hsa04940 | 2.69E-14 | 1.78E-12 |
| Graft-versus-host disease | path:hsa05332 | 4.34E-13 | 2.39E-11 |
| Viral myocarditis | path:hsa05416 | 2.44E-12 | 1.15E-10 |
| Asthma | path:hsa05310 | 1.43E-11 | 5.89E-10 |
| Staphylococcus aureus infection | path:hsa05150 | 2.54E-11 | 8.87E-10 |
| Herpes simplex infection | path:hsa05168 | 2.69E-11 | 8.87E-10 |
| Intestinal immune network for IgA production | path:hsa04672 | 1.04E-10 | 3.13E-09 |
| Phagosome | path:hsa04145 | 7.98E-10 | 2.19E-08 |
| Alcoholism | path:hsa05034 | 1.24E-09 | 3.14E-08 |
| Inflammatory bowel disease (IBD) | path:hsa05321 | 2.62E-09 | 6.18E-08 |
| Cell adhesion molecules (CAMs) | path:hsa04514 | 3.27E-09 | 7.19E-08 |
| Epstein-Barr virus infection | path:hsa05169 | 6.76E-09 | 1.39E-07 |
| Th1 and Th2 cell differentiation | path:hsa04658 | 1.12E-07 | 2.17E-06 |
| Leishmaniasis | path:hsa05140 | 1.34E-07 | 2.46E-06 |
| Human T-cell leukemia virus 1 infection | path:hsa05166 | 1.54E-07 | 2.67E-06 |
| Viral carcinogenesis | path:hsa05203 | 2.96E-07 | 4.89E-06 |
| Th17 cell differentiation | path:hsa04659 | 5.34E-07 | 8.39E-06 |
| Rheumatoid arthritis | path:hsa05323 | 8.73E-07 | 1.31E-05 |
| Toxoplasmosis | path:hsa05145 | 9.28E-07 | 1.33E-05 |
| Hematopoietic cell lineage | path:hsa04640 | 1.76E-06 | 2.42E-05 |
| Influenza A | path:hsa05164 | 9.36E-06 | 1.24E-04 |
| Tuberculosis | path:hsa05152 | 7.67E-05 | 9.73E-04 |
| Human cytomegalovirus infection | path:hsa05163 | 2.08E-03 | 2.54E-02 |

* Qiu Y-H, Deng F-Y, Li M-J, Lei S-F. Identification of novel risk genes associated with type 1 diabetes mellitus using a genome-wide gene-based association analysis. J Diabetes Investig. 2014. doi:10.1111/jdi.12228

** (adjusted p-value ≤ 0.05)
